# Essential Oils of *Gardenia jasminoides* J. Ellis and *Gardenia jasminoides* f. *longicarpa* Z.W. Xie & M. Okada Flowers: Chemical Characterization and Assessment of Anti-Inflammatory Effects in Alveolar Macrophage

**DOI:** 10.3390/pharmaceutics14050966

**Published:** 2022-04-29

**Authors:** Nan Zhang, Ying Bian, Lei Yao

**Affiliations:** 1 School of Design, Shanghai Jiao Tong University, 800 Dong Chuan Road, Shanghai 200240, China; fxzwzhangnan@sjtu.edu.cn; 2 Aromatic Plant R&D Center, Shanghai Jiao Tong University, 800 Dong Chuan Road, Shanghai 200240, China; 3 School of Agriculture and Biology, Shanghai Jiao Tong University, 800 Dong Chuan Road, Shanghai 200240, China; ybian1@stu.mcphs.edu

**Keywords:** *Gardenia jasminoides*, essential oil, alveolar macrophage, anti-inflammatory, nitric oxide, TNF-α, PGE2

## Abstract

Alveolar macrophage is the predominant cell type in the lung and is thought to be the major target for anti-inflammatory therapy in chronic obstructive pulmonary disease (COPD). Aromatherapy using natural essential oils with anti-inflammatory effects for inhalable administration is a potential complementary and alternative therapy for COPD treatment. The *Gardenia jasminoides* flower is famous for its fragrance in East Asia and is used for treating colds and lung problems in folk medicine. Therefore, in the present study, flower essential oils from two main medicinal gardenia varieties (*G. jasminoides* J. Ellis and *G. jasminoides f. longicarp*a Z.W. Xie & M. Okada) were extracted by hydro-distillation, and their chemical components were analyzed by GC-MS. The anti-inflammatory effects of the two essential oils and their main ingredients were further studied on lipopolysaccharide (LPS)-induced models in murine alveolar macrophages (MH-S). The results indicated that the chemical constituents of the two gardenia varieties were quite different. Alcohol accounted for 53.8% of the *G. jasminoides* essential oil, followed by terpenes (16.01%). Terpenes accounted for 34.32% of the *G. jasminoides* f. *longicarpa* essential oil, followed by alcohols (19.6%) and esters (13.85%). Both the two gardenia essential oils inhibited the LPS-induced nitric oxide (NO) release and reduced the production of tumor necrosis factor-α (TNF-α) and prostaglandin E2 (PGE2) in the MH-S cells. Linalool and α-farnesene dose-dependently reduced the NO release in the MH-S cells. Linalool and α-farnesene did not affect the PGE2 production but regulated the expression of TNF- α. In addition to linalool and α-farnesene, other components in the gardenia flower essential oils appeared to be able to act as anti-inflammatory agents and influence the PGE2 pathway.

## 1. Introduction

Chronic obstructive pulmonary disease (COPD) is a globally increasing disease characterized by progressive airway obstruction and associated with chronic inflammation [1]. It was identified in 2020 as the third most frequent cause of death, with a substantial financial burden for society [2]. Alveolar macrophage (AM) is the predominant cell type in the lung and is thought to be the major target for anti-inflammatory therapy [3]. AM activation and the release of the cytokine play important roles in the pathogenesis of COPD [4]. Upon activation by harmful stimuli such as cigarette smoke and bacterial infections, AMs release inflammatory mediators including nitric oxide (NO), prostaglandin E2 (PGE2), and pro-inflammatory cytokines such as tumor necrosis factor-α (TNF-α) and interleukin-6 (IL-6). Excessive accumulation of the above mediators would destroy the lung tissue and lead to respiratory dysfunction [5]. Currently, pharmacological therapy in COPD treatment is divided into bronchodilators and anti-inflammatory agents. Corticosteroids and β_2_ agonist bronchodilators are wildly used for COPD treatment [6]. A certain number of COPD patients are resistant to corticosteroid treatment. One previous study showed that lipopolysaccharide (LPS)-induced IL-8, GM-CSF, MCP-1, and MMP-9 release from alveolar macrophages was partially resistant to corticosteroids in COPD, while resveratrol provided an alternative [7]. Therefore, more effective anti-inflammatory drugs and combined treatment are required [8].

Aromatherapy using natural essential oils for inhalable administration might be a potential complementary and alternative therapy for COPD treatment. Many essential oils from aromatic plants such as *Margotia gummifera* (Desf.) Lange [9], *Schinus areira* L. [10], *Lavandula angustifolia* Mill. [11], *Matricaria chamomilla* L. [12], and *Cordia verbenacea* D.C. [13] have been proven to have lung-related anti-inflammatory effects. Limonene [14], α-pinene [15], linalyl acetate [16], linalool [17], carvacrol [18], borneol [19], (-)-α-bisabolol [20], 1,8-cineole [21], citral [22], and trans-cinnamaldehyde [23] were thought to be the anti-inflammatory active components in essential oils. Intraperitoneal injection of linalool could inhibit NF-κ B activity to alleviate lung inflammation induced by cigarette smoke [17]. Oral or aerosol intake of α-humulene showed a significant anti-inflammatory effect in an airway allergic inflammation mice model [24]. In some COPD-related models, essential oils or their main chemical components have been proven to have anti-inflammatory effects. *Zataria multiflora* Boiss. and its volatile constituent carvacrol showed preventive effects on lung inflammation changes and oxidative stress in an animal model of COPD [25]. Cannabis oil affected the expression of specific airway epithelial cell genes that could modulate pro-inflammatory or Th1 processes in COPD [26]. Linalyl acetate prevented three related factors of vascular damage in COPD-like and hypertensive rats [27]. In another clinical trial, concomitant therapy with cineole reduced exacerbations as well as dyspnea and improved lung function and health status [28].

*Gardenia jasminoides* J. Ellis (Rubiaceae) is native to subtropical regions of East Asia. It is a very famous fragrant flower and is used as a vegetable for soups and cold salads in China. In folk medicine, the flowers, roots, and fruits of gardenia are often used as medicines. ‘Shanzhizi’ (*G. jasminoides*) and ‘Shuizhizi’ (*G. jasminoides* f. *longicarpa* Z.W. Xie & M. Okada) are the most common gardenia cultivars. They are widely distributed and cultivated in most of the provinces and regions south of the Yangtze River. They have been treated separately in herbal monographs in China. The fruit of *G. jasminoides* has been used in traditional Chinese medicine for a long time. Several studies have proven its potential neuroprotective effects [29], anti-inflammatory effects [30], and memory-enhancing capacity [31]. The fruit of *G. jasminoides* f. *longicarpa* is an important natural yellow-pigmented raw material that is widely used in the food and chemical industries [32]. At present, only in the Fuding area of Fujian province in China, there are 4500-hectare gardenia planting bases, with an annual flower output of approximately 3000 kg per hectare. In 2020, there was an annual output of approximately 10,000 tons of gardenia flowers, and there are more than five local essential oil processing factories in this region. Based on such a large planting scale and flower yield, the development and application of the medicinal function of *G. jasminoides* essential oil will play a positive role in enhancing its industrial value. Many studies have pointed out that the plant essential oil composition is related to many factors. Planting geographical region factors might lead to a difference in essential oil composition [33]. Different cultivars of the same species might have different essential oil compositions [34]. Moreover, harvest year [35], harvest date [36], and fertilizers [37] might affect the quality and yield of the essential oil. The difference in the chemical composition of essential oil mentioned above might affect its medicinal value.

Compared with the aromaticity of the flower, the potential effects of the volatile components of the flower have received less attention. At present, only very little research focuses on the potential effects of the volatile components of the flower. According to a previous study, linalool, α-terpineol, cis-3-hexenyl tiglate, and α-farnesene are the main volatile components of *G. jasminoides* [38]. There is still no study that has reported the volatile components of *G. jasminoides* f. *longicarpa*, which is also a very important herbal plant with a large planting area. Only a few papers have reported on the antibacterial activities and anti-anxiety effects of the *G. jasminoides* flower essential oil [38,39].

It is recorded in the Chinese materia medica of ‘Southern Yunnan’ that ‘for the treatment of cold with phlegm and fire in the lung, use three Gardenia flowers and a little honey, which are fried and then taken together’ [40]. This indicates that the gardenia flower has a therapeutic effect on respiratory diseases. At present, the research on the anti-inflammatory effect of the gardenia mainly focuses on the iridoid macromolecules in fruits [41], and the mechanisms of active gardenoside and genipin have been verified [42,43]. Gardenia leaf essential oil, which mainly contained 49.2% pentadecanal and 12.3% geranial, showed an anti-inflammatory effect in rats [44]. The anti-inflammatory effect of the volatile components of the gardenia flower has not been reported yet. It is well known that small-molecular compounds have better transdermal absorption and have great advantages in external use. It also has application advantages in inhalation administration. Therefore, the anti-inflammatory effect of *G. jasminoides* aroma components has its research value.

In this study, essential oils from *G. jasminoides* and *G. jasminoides* f. *longicarpa* flowers were extracted by hydro-distillation and their chemical components were analyzed by gas chromatography–mass spectrometry (GC-MS). The anti-inflammatory effects of the two essential oils and their main ingredients were further studied on LPS-induced models in murine alveolar macrophages (MH-S).

## 2. Materials and Methods

### 2.1. Plant Material, Essential Oil Preparation, and Chemicals

**Figure 1:**
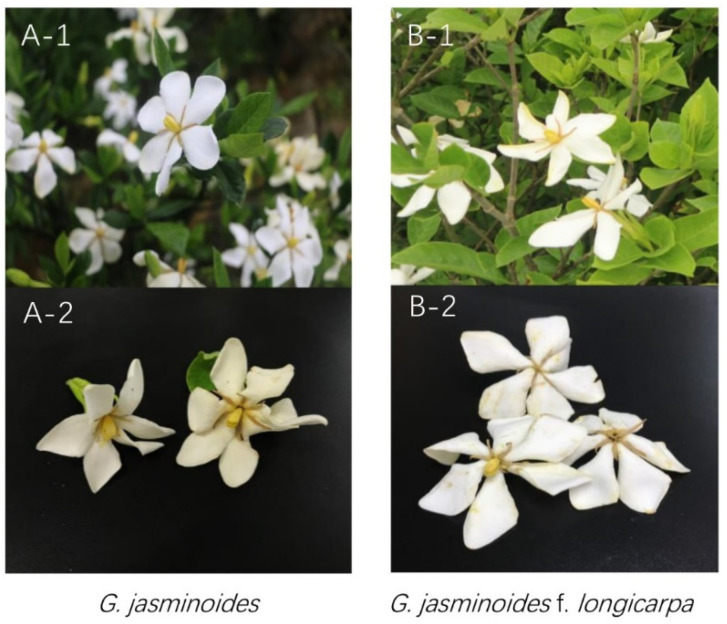
The flower of *G. jasminoides* (**A**) and *G. jasminoides* f. *longicarpa* (**B**). (**A**-**1**,**B**-**1**), the naturally growing flowers in the planting base in Zhejiang province. (**A**-**2**,**B**-**2**), the fresh flower used for essential oil extraction in the lab.

Fresh flowers of *G. jasminoides* and *G. jasminoides* f. *longicarpa* (Figure 1) were collected from May to June from Zhejiang province of China (Pingyang County, 27°18′ N, 120°30′ E), provided by a local planting company (Zhejiang Xingguang Agricultural Development Co., Ltd. Wenzhou, China). The local climate type was a subtropical monsoon climate and the soil was slightly acidic. The essential oils of *G. jasminoides* (GJE) and *G. jasminoides* f. *longicarpa* (GJLE) were extracted from the fresh flower by hydro-distillation using the method reported in a previous study [39]. One kilogram of flowers and 3 L of water were added to a 5 L distillation device (Clevenger type), followed by ultrasonic treatment for 20 min and constant-temperature distillation for 4 h. The upper light-yellow-colored essential oil in the oil–water separator was collected. In total, 0.07 g and 0.02 g of essential oil could be extracted on average from 1 kg of fresh *G. jasminoides* and *G jasminoides* f. *longicarpa* flowers, respectively. Chemical compounds including linalool (PubChem SID: 24901213) and α-farnesene (PubChem SID: 24901903) were purchased from Sigma-Aldrich (St. Louis, MO, USA).

### 2.2. Identification of the Constituents of the Essential Oil

The essential oil was diluted using a solution of ethanol and n-hexane (1:1, *v/v*). GC-MS (Agilent 7890B-5977A) was used to analyze the components of the essential oils; the instrument was equipped with a methylpolysiloxane nonpolar column (HP-5MS: 30 m × 0.25 mm × 0.25 μm). The GC was operated under the following conditions: carrier gas, helium (1 mL/min); split rate, 10:1; column temperature, 50 °C, lasting for 10 min, 50 °C to 220 °C at 4 °C/min, then lasting for 10 min, 220 °C to 280 °C at 20 °C/min, then lasting for 3 min. The MS operating parameters were as follows: inlet line temperature, 280 °C; source temperature, 230 °C; mass spectra electron impact, 70 eV. Individual components were identified from the mass spectral library (NIST14).

### 2.3. Cell Culture and Chemical Treatment

MH-S (BeNa Culture collection, BNCC, Xinyang, China) were cultured in RPMI 1640 culture medium (HyClone, Logan, UT, USA) supplemented with 10% FBS, 100 U/mL penicillin, 100 mg/mL streptomycin (Gibco, Madrid, Spain) at 37 °C in 95% humidified air containing 5% CO_2_.

The essential oil, or the compound, was mixed with DMSO in the ratio of 1:1, and then was added into the complete medium to prepare the mother liquor, and it was stored at 4 °C. For use in the treatment of cells, the mother liquor was diluted into the required concentration gradient with a complete medium.

### 2.4. Cell Viability Assay

Effects of essential oils or compounds on the proliferation and viability of MH-S cells were analyzed using the Cell Counting Kit-8 (CCK8) assay. MH-S cells were cultured in a 96-well microplate at the concentration of 2.5–3 × 10^5^ cells per well and incubated in an incubator with an atmosphere of 5% CO_2_ at 37 °C for 24 h. Except for the control wells, the remaining wells were added to the culture medium containing gardenia flower essential oils, linalool, or α-farnesene. Final concentrations of essential oils or compounds were 0.1, 1, 10, 100, 200, 500, and 1000 μg/mL. Then, cells were cultured for another 24 h. The ODs were measured according to the protocol of the CCK8 assay kit (Shanghai Biyuntian Biological Co., Ltd., Shanghai, China).

### 2.5. Measurement of Nitric Oxide Levels, PGE2, and TNF-α

Cells were incubated with various concentrations of essential oil (0.1–200 μg/mL), or chemicals such as linalool (10–500 μg/mL) and α-farnesene (10–500 μg/mL), for 1 h, and then incubated in the presence or absence of 10 μL LPS for 24 h. The conditioned medium was collected for analysis. According to a pre-experiment (Supplementary Figure S1), 2 μg/mL was selected as the final concentration of LPS.

The nitric oxide (NO) concentration in the culture medium was measured by a Griess reaction test. In a 96-well microplate, 100 μL of the Griess reagent (Beyotime, Shanghai, China) was mixed with an equal volume of cell supernatant, the optical density at 540 nm was measured, and the concentration of nitrite was calculated according to the standard curve generated from known concentrations of sodium nitrite. The accumulated PGE2 and TNF-α in the culture medium were measured using an ELISA Kit (Beyotime, Shanghai, China) according to the manufacturer’s instructions.

### 2.6. Statistical Analysis

Data were expressed as mean ± SD. Mean group values were compared using one-way ANOVA for normally distributed variables. Post hoc comparisons on normally distributed variables were performed using the Duncan test. A value of *p* < 0.05 was considered statistically significant.

## 3. Result

### 3.1. The Main Constituents of the Gardenia Flower Essential Oils

The essential oils of gardenia flowers were analyzed by GC-MS. There were 18 components with relative content greater than 0.5% in the GJE, accounting for 85.34% of the total (shown in Table 1). The main aroma components were linalool (34.65%) α-farnesene (10.24%), α-terpineol (6.27%), geraniol (5.79%), cembrene A (5.77%), cis-3-hexenyl tiglate (3.13%), and tau-cadinol (1.77%).

There were 27 components with relative content greater than 0.5% in the essential oil of GJLE, accounting for 72.64% of the total (shown in Table 1). The main aroma components were α- farnesene (32.45%), cis-3-hexenyl tiglate (8.02%), linalool (6.56%) α-terpineol (2.40%), tau-cadinol (2.40%), geraniol (1.93%), and 3-hexen-1-ol, benzoate, (Z)- (1.76%).

Long-chain alkane compounds, such as tricosane and pentacosane, could also be detected, and accounted for more than 5% of the total compounds in both of the two essential oils. This might be the reason for the phenomenon wherein both of the two essential oils were solid at room temperature.

**Figure 2:**
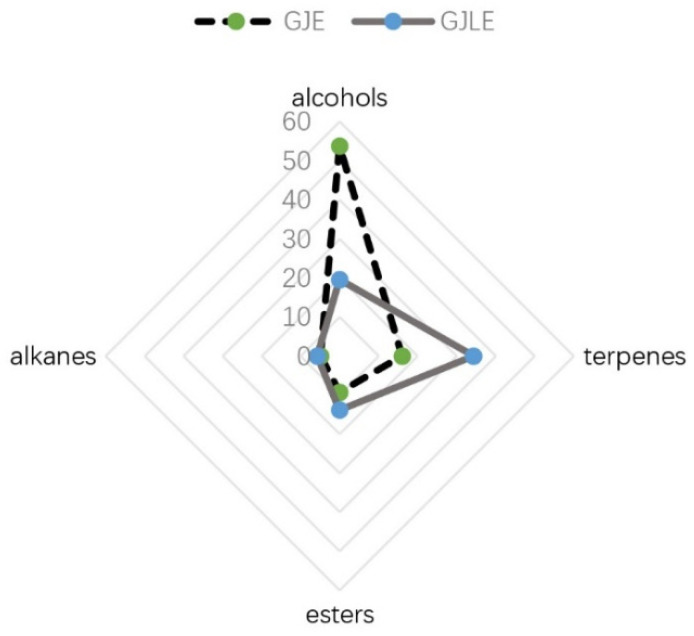
The chemical composition of the two gardenia essential oils. Data represent the sum of compounds with a relative content higher than 0.5% in the oils with the same chemical type.

The proportion of different types of compounds in the two essential oils was different (Figure 2). In all components with relative content greater than 0.5%, alcohols accounted for 53.80% in GJE, followed by terpenes (16.01%), esters (9.30%), and alkanes (4.89%). Terpenes accounted for 34.32% in GJLE, followed by alcohols (19.6%), esters (13.85%), and alkanes (5.75%).

### 3.2. Effects of Gardenia Essential Oils on the Viability of MH-S Cells

**Figure 3:**
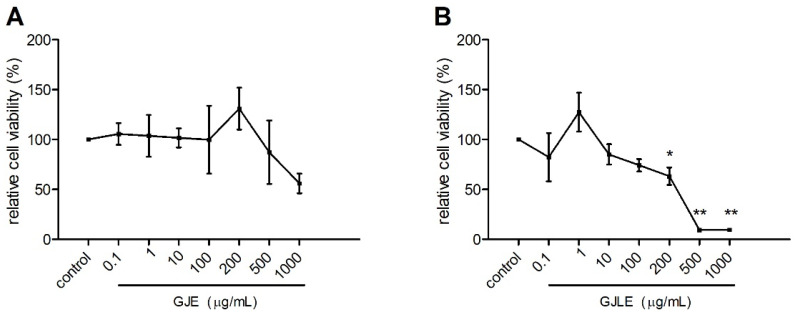
Effects of *G. jasminoides* (**A**) and *G. jasminoides* f. *longicarpa* (**B**) flower essential oils on the viability of MH-S cells. Values represent the mean ± SD from three independent experiments, * *p* < 0.05 and ** *p* < 0.01 vs. the control treatment; one-way ANOVA was used, and Duncan test was used for post hoc analysis.

In the intervention group of GJE, the cell viability increased first and then decreased with the increase in the concentration of the GJE (Figure 3A). However, there was no significant difference compared with the control treatment (*p* > 0.05). In the intervention group of GJLE, the cell viability first increased and then decreased with the increase in the content of essential oil (Figure 3B). Treatments with 200, 500, and 1000 μg/mL of GJLE significantly inhibited the cells’ viability compared with the control treatment (*p* < 0.05). According to these results, 0.1–200 μg/mL of GJE and GJLE were used in the following studies.

### 3.3. Effects of Gardenia Essential Oils on the NO, TNF-α, and PGE2 Secretion of MH-S Cells

**Figure 4:**
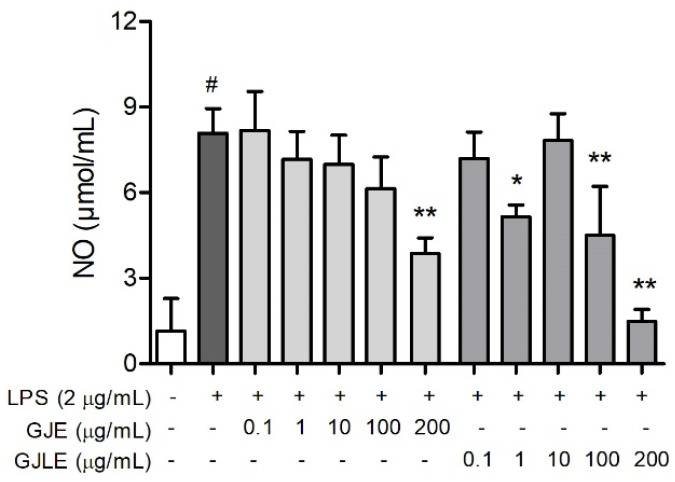
Effect of *G. jasminoides* and *G. jasminoides* f. *longicarpa* flower essential oils on the NO secretion of MH-S cells. Values represent the mean ± SD from three independent experiments, * *p* < 0.05 and ** *p* < 0.01 vs. the control treatment group with LPS; # means *p* < 0.05 vs. the control treatment group without LPS. One-way ANOVA was used, and Duncan test was used for post hoc analysis.

LPS (2 μg/mL) treatment significantly increased the NO release in the MH-S cells (Figure 4). The inhibitory effect of GJLE on NO release was stronger than that of GJE. Compared with the LPS + control treatment, GJLE at 1, 100, and 200 μg/mL could significantly reduce the NO release (*p* < 0.05) in the LPS-treated cells. GJE inhibited LPS-induced NO release in a dose-dependent manner. However, only 200 μg/mL of GJE showed a significant effect compared with the control treatment (*p* < 0.01).

**Figure 5:**
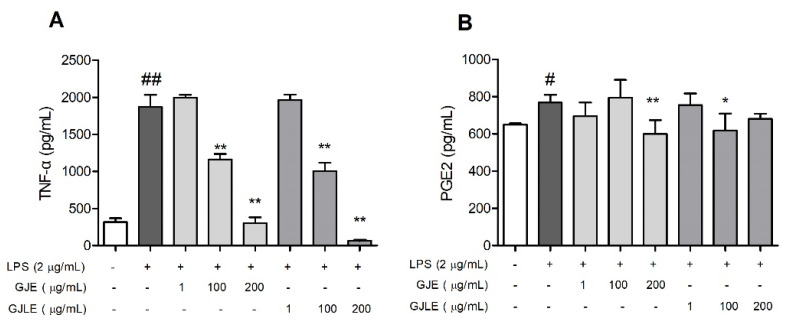
Effects of *G. jasminoides* (**A**) and *G. jasminoides* f. *longicarpa* (**B**) flower essential oils on the expression of TNF-α and PGE2 in MH-S cells. Values represent the mean ± SD from four independent experiments, * *p* < 0.05 and ** *p* < 0.01 vs. the control treatment group with LPS; # means *p* < 0.05 and ## means *p* < 0.01 vs. the control treatment group without LPS. One-way ANOVA was used, and Duncan test was used for post hoc analysis.

Compared with the control treatment without LPS, LPS significantly increased the expression of the pro-inflammatory cytokine TNF-α in the MH-S cells (*p* < 0.01). Treatment with gardenia essential oil dose-dependently reduced the TNF-α content in the cell culture medium (Figure 5A). At 100 and 200 μg/mL, both GJLE and GJE significantly reduced the expression of TNF-α compared to the LPS + control treatment (*p* < 0.01). LPS also significantly increased the expression of the inflammatory mediator PGE2 in the MH-S cells (*p* < 0.05). GJE (200 μg/mL) and GJLE (100 μg/mL) significantly reduced the expression of PGE2 compared to the LPS + control treatment (*p* < 0.05).

### 3.4. Anti-Inflammatory Effects of Two Main Ingredients of Gardenia Essential Oils

Linalool and α-farnesene were the two main ingredients in gardenia essential oils, while the content of the two compounds in GJE and GJLE was quite different. According to the previous results, the two gardenia essential oils differed in their ability to inhibit NO release. Therefore, the anti-inflammatory effects of the two main compounds were further evaluated separately.

**Figure 6:**
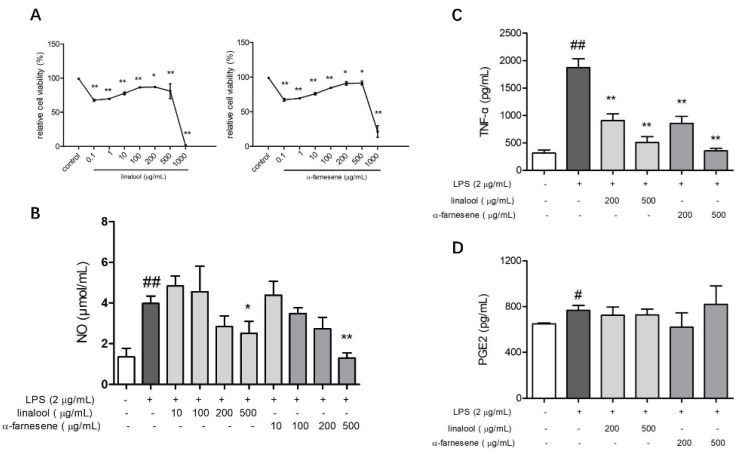
Effects of linalool and α-farnesene on the viability (**A**), NO secretion (**B**), and expression of TNF-α (**C**) and PGE2 (**D**) in MH-S cells. Values represent the mean ± SD from three or four independent experiments, * *p* < 0.05 vs. the control treatment, ** *p* < 0.01 vs. the control treatment in Figure 4A; * *p* < 0.05 and ** *p* < 0.01 vs. the control treatment group with LPS, # *p* < 0.05 and ## *p* < 0.01 vs. the control treatment group without LPS in Figure 4B–D. One-way ANOVA was used, and Duncan test was used for post hoc analysis.

The two compounds showed similar effects on the viability of MH-S cells. The cell viability decreased when treated with 0.1 μg/mL of linalool or α-farnesene, and then increased with the increase in the compound concentration at the range of 1–200 μg/mL (*p* < 0.05), but the cell viability was still lower than in the control treatment. Both linalool and α-farnesene significantly inhibited the cell viability at 1000 μg/mL (Figure 6A). Thus, 10–500 μg/mL concentrations were used in the following studies.

There was a dose-dependent decrease in the NO release induced by LPS when treated with linalool or α-farnesene at 10–500 μg/mL in the MH-S cells (Figure 6B). Compared with the control treatment, the two compounds significantly reduced NO content at the concentration of 500 μg/mL (*p* < 0.05). According to the CCK8 results, the inhibitory effect on NO might not be caused by apoptosis. Both of the two compounds significantly inhibited the expression of TNF-α at 200 and 500 μg/mL (*p* < 0.01) but showed no effect on the expression of PGE2 (*p* > 0.05) (Figure 6C,D).

## 4. Discussion

In the flavor and fragrance or aromatherapy industries, essential oils are often classified according to the main volatile components. Many plant oils are often chemically diverse due to factors such as species and origin. In some of our previous studies, it has been found that there might be great differences in the volatile components of aromatic plants belonging to different varieties [45]. For example, the chemical compositions of essential oils extracted from different varieties of lavender varied greatly. Some of them mainly contained linalool and linalyl acetate, and some of them mainly contained 1,8-cineole or limonene [46]. In the present study, the chemical constituents of gardenia essential oil from two different varieties were also quite different. Although linalool and α-farnesene were the main components, there were great differences in content. The proportion of different types of compounds in the two essential oils was different. Alcohols accounted for 53.8% in GJE, followed by terpenes (16.01%). Terpenes accounted for 34.32% in GJLE, followed by alcohols (19.6%) and esters (13.85%). Whether such differences in chemical composition cause functional differences is worth exploring.

In the present study, the results showed that GJE had no significant cytotoxicity to MH-S cells in the concentration range of 0.1–1000 μg/mL, while GJLE had significant cytotoxicity at the concentration of 200 μg/mL and above. In the following study of NO, PGE2 and TNF-α, 200 μg/mL and lower concentrations were used in the oil treatment. GJLE at 200 μg/mL only suppressed the expression of TNF-α, but not PGE2, which indicated that its effect on NO and TNF-α regulation might not be the consequence of cell viability decreases. Such cytotoxicity differences might due to the chemical differences of the two essential oils. However, the main components such as linalool and α-farnesene might not be the reason for this phenomenon. These two components showed similar cytotoxicity in the concentration range used (0.1–1000 μg/mL), but it was only at 1000 μg/mL that the cell viability decreased greatly.

LPS, commonly called endotoxin, is involved in sepsis and septic shock syndrome in human infections due to Gram-negative pathogenic bacteria. LPS stimuli macrophages release inflammatory mediators including NO. Extensively used in preclinical investigations, the intrapulmonary exposure of LPS triggers the production of inflammatory mediators [47]. In the present study, both of the two gardenia essential oils could inhibit the NO release in the MH-S cells. The inhibitory effect of GJLE on NO release was stronger than that of GJE. Linalool and α-farnesene dose-dependently reduced the NO release of the MH-S cells in the range of 10–500 µg/mL. α-Farnesene, the main component of GJLE, was more efficient than linalool at 500 µg/mL. This might be one of the reasons that GJLE showed a stronger inhibitory effect on NO release than GJE.

In previous studies, linalool had been reported to relieve edema after carrageenin administration [16], exert preventive effects against the influx of inflammatory cells and mucus hypersecretion in the lung tissues [48], and inhibit the biting response induced by IL-1β and TNF-α in mice [49]. In the present study, gardenia flower essential oil and the two main compounds inhibited TNF-α release induced by LPS, while only the essential oils showed some effects on the inhibition of PGE2 expression. This result was consistent with a previous study wherein linalool reduced NO release but failed to inhibit PGE2 release in J774.A1 cells [50]. PGE2 is a potent pro-survival mediator in neutrophils that is increased in COPD and might contribute to neutrophilic accumulation in COPD lungs. Essential oils from *Cinnamomum longepaniculatum* (Gamble) N. Chao ex H. W. Li. leaves (hydrodistillation, Yibin city of Sichuan province, China) [51], *Hyptis pectinate* (L.) Poit leaves (hydrodistillation, northeastern Brazil) [52], and *Mentha piperita* L. leaves (hydrodistillation, Jinan, China) [53] have been reported to be effective in reducing the histological PGE2 in mice or the LPS-induced PGE2 in RAW 264.7 macrophages. As a complex of compounds, the essential oil’s anti-inflammatory activities might involve multiple signaling pathways. The present results are consistent with some previous research results. In one previous study, it was reported that the anti-inflammatory activity of the essential oil of *Cinnamomum osmophloeum* Kaneh leaves (hydrodistillation, central Taiwan) on PGE2 was not attributable to the major constituents such as caryophyllene oxide and L-bornyl acetate, but might be attributable to the effects of minor constituents or synergetic effects among the constituents [54]. In some other studies, the main compounds of essential oils showed a good effect. Patchouli alcohol, the main compound that was isolated from *Pogostemon cablin* (Blanco) Benth., decreased the production of TNF-α, IL-6, NO, and PGE2 in LPS-stimulated RAW 264.7 cells [55]. In the present study, the anti-inflammatory effect of linalool and α-farnesene did not affect the production of PGE2 but regulated the expression of TNF-α. It is speculated that, in addition to linalool and α-farnesene, other components in the gardenia flower essential oils might exert important anti-inflammatory effects and act on the arachidonic acid metallic pathway.

## 5. Conclusions

Both the two gardenia essential oils showed anti-inflammatory effects. GJE and GJLE inhibited the LPS-induced NO release and reduced the production of TNF-α and PGE2 in the MH-S cells. Linalool and α-farnesene, which were the two main components of the gardenia flower essential oils, reduced the NO release and TNF-α secretion while showing no effect on the expression of PGE2. In addition to linalool and α-farnesene, other components in the gardenia flower essential oils are speculated to play anti-inflammatory effects and act on the PGE2-related pathway.

## Figures and Tables

**Table 1:** The main constituents of the essential oils of the two gardenia flowers.

No.	Retention Time (min)	Name	Peak Area (%)	
			GJE	GJLE
1	14.35	Linalool	34.65	6.56
2	17.19	α-Terpineol	6.27	2.40
3	18.41	Nerol	1.96	0.64
4	19.66	Geraniol	5.79	1.93
5	21.78	cis-3-Hexenyl tiglate	3.13	8.02
6	21.99	Hexyl tiglate	2.39	1.23
7	28.00	α-Farnesene	10.24	32.45
8	28.34	Octyl (E)-2-methylbut-2-enoate	0.70	0.52
9	29.50	(+)-trans-Nerolidol	-	0.53
10	29.66	3-Hexen-1-ol, benzoate, (Z)-	-	1.76
11	29.82	Benzoic acid, hexyl ester	-	0.66
12	29.92	(3E,7E)-4,8,12-Trimethyltrideca-1,3,7,11-tetraene	-	0.90
13	31.79	tau-Cadinol	1.77	2.40
14	32.17	α-Cadinol	0.68	0.74
15	32.30	α-Terpinyl acetate	1.19	-
16	33.51	Geranyl angelate	1.89	0.55
17	36.57	8-Hydroxylinalool	1.67	1.23
18	40.67	Cembrene A	5.77	0.97
19	40.86	Verticillol	1.01	0.97
20	41.55	n-Hexadecanoic acid	1.33	5.70
21	42.10	3,7,11,15-Tetramethylhexadeca-1,6,10,14-tetraen-3-ol	-	1.17
22	45.10	Benzoic acid, [(E,E)-3,7,11-trimethyl-2,6,10-dodecatrien-1-yl] ester	-	1.11
23	45.87	trans-Geranylgeraniol	-	1.03
24	45.69	9,12-Octadecadienoic acid (Z,Z)-	-	1.44
25	48.44	Tricosane	1.93	0.90
26	52.66	Pentacosane	2.96	3.68
27	56.37	Heptacosane	-	0.53
28	58.77	Squalene	-	0.64
Sum			85.33	72.64

The table lists those components whose peak area is >0.5% of the total peak area; “-” means not detected or the peak area <0.5%.

## Data Availability

Data are contained within the article.

## Supplementary Materials

The following are available online at www.mdpi.com/article/10.3390/pharmaceutics14050966/s1, Figure S1: The effect of LPS dose on the NO release in MH-S cells. Values represent the mean ± S.D., ^##^ *p* < 0.01 vs. the control treatment group without LPS. One-way ANOVA was used, and Duncan test was used for post hoc.
