# Supplementary material for: Essential Oils of Gardenia jasminoides J. Ellis and Gardenia jasminoides f. longicarpa Z.W. Xie & M. Okada Flowers: Chemical Characterization and Assessment of Anti-Inflammatory Effects in Alveolar Macrophage
Source: Pharmaceutics. 2022 Apr 29;14(5):966. doi: 10.3390/pharmaceutics14050966 (PMC9145545; doi:10.3390/pharmaceutics14050966)
Supplement: Supplementary file 1 [file pharmaceutics-14-00966-s001.zip › pharmaceutics-1671940-supplementary.pdf]

pharmaceutics

Supplementary Materials: Essential Oils of *Gardenia jasminoides* J. Ellis and *Gardenia jasminoides* f. *longicarpa* Z.W. Xie & M. Okada Flowers: Chemical Characterization and Assessment of Anti-Inflammatory Effects in Alveolar Macrophage

Nan Zhang, Ying Bian and Lei Yao *

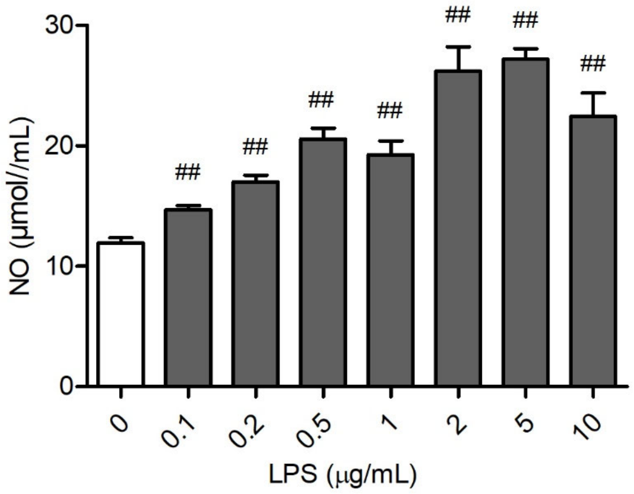

Figure S1. The effect of LPS dose on the NO release in MH-S cells. Values represent the mean ± S.D., ## $p < 0.01$ vs. the control treatment group without LPS. One-way ANOVA was used, and Duncan test was used for post hoc.
